# Developing informed consent materials for non-English-speaking participants: An analysis of four professional firm translations from English to Spanish

**DOI:** 10.1177/1740774518801591

**Published:** 2018-10-08

**Authors:** Kathleen Marie Brelsford, Ernesto Ruiz, Laura Beskow

**Affiliations:** 1 Center for Biomedical Ethics and Society, Vanderbilt University Medical Center, Nashville, TN, USA; 2 Department of Family & Community Medicine, Meharry Medical College, Nashville, TN, USA

**Keywords:** Translation, Spanish, informed consent, cross-cultural, readability, non-English

## Abstract

**Background/Aims:**

An increasing body of research is being conducted with non-English-speaking subjects. Study-related materials, including those essential for obtaining informed consent, must often be translated from English into other languages. In this study, we sought to examine the types of issues that may arise when consent materials are translated from English to Spanish.

**Methods:**

Drawing on expertise from five individuals associated with our research team, four of whom are native Spanish speakers of different dialects of Spanish, we crafted translations of our own consent materials for biobanking using a rigorous, multi-step process involving both forward and back translation. We then systematically compared our translations to those produced by four professional translation firms to identify potential concerns in our own and the professional translations.

**Results:**

We identified three primary types of problems of relevance for researchers conducting studies where translation of written information is required. These included nonequivalent registers (in particular, the introduction of more complicated language), errors of omission (reducing the clarity of the information), and changes that altered the substantive meaning of the information.

**Conclusion:**

Our findings highlight the importance of working with translators who not only possess “textbook” knowledge of both languages but also an appreciation of the sociocultural factors that affect how people interpret and understand meaning. Moreover, translators who have a basic understanding of research are more likely to accurately convey essential research concepts. We describe a series of steps researchers can take that may help to improve the quality of translated materials.

## Introduction

For research involving human subjects, informed consent is required (with limited exceptions), both ethically and legally. As part of this requirement, study information must be presented “in language understandable to the subject.”^1–3^ The Office of Human Research Protections strongly encourages that non-English-speaking subjects be provided with written consent materials in their own language.^4^ Creating consent materials in languages other than English, and ensuring that they are understandable to participants, can be a significant challenge.

In general, consent documents are often written using complex and technical language and empirical analyses continue to reveal major limitations in subject comprehension.^5–16^ As one way to confront comprehension issues, some researchers have invested significant effort to empirically develop and test simpler consent materials.^17–24^ While these efforts cannot solve all comprehension problems, they are an important step in improving the informed consent process.^25^

Unfortunately, such efforts are often limited to development of English materials. The challenges to providing non-English-speaking subjects with informative and understandable consent materials are compounded when information must be translated and adapted from English. Rather than a straightforward, mechanical process in which there exists a word-for-word correspondence between two languages, translation is a craft that heavily relies on the translator’s skills and knowledge.^26,27^

As part of a National Institutes of Health (NIH)-funded study on simplifying informed consent for biobanking, we sought to produce high-quality translations of three empirically developed consent-related documents from English to Spanish. To that end, we undertook a rigorous process by which multiple native Spanish speakers associated with our research team collaborated to translate the materials. We then hired four professional firms to undertake independent translations in order to examine the types of errors and issues that can arise when a more typical translation process is used.

## Methods

Our goal was to produce high-quality Spanish translations of three consent-related documents originally developed in English: a 6-page consent form explicitly designed to closely resemble biobank consent forms in actual use, a 3-page simplified biobank consent form, and a 15-question consent comprehension quiz with accompanying explanations of the correct answers. All three documents were empirically developed in previous Aims of the grant.^25^

### Description of the research team’s translation process

*Step 1—Initial forward translation to Spanish.* Two native Spanish speakers (Argentine and Ecuadorian) who routinely consent Spanish speakers to participate in biobank research provided the initial forward translations. One individual generated each of the initial Spanish translations. The second then closely reviewed each translation against the original English version to identify potential problems (e.g. regionally specific language; failure to translate a concept; change of meaning; discordance in tone, formality, or reading level). These two individuals then discussed each issue to reach a resolution.

*Step 2—Back translation to English.* One of the co-authors (E.R., native Costa Rican Spanish speaker), who had not seen the original English documents, then translated the Spanish materials produced in Step 1 back to English.

*Step 3—Comparison of back translation to original English materials.* Two co-authors (E.R. and K.M.B., a second language Spanish speaker) then used the “compare” feature in Word to examine differences between the original English materials and their corresponding back translations produced in Step 2. For each comparison document generated, the co-authors independently categorized each discrepancy as either acceptable or problematic. Acceptable discrepancies included instances in which variations between the original and back-translated English forms did *not* alter the meaning but rather were expected effects of the translation process, such as variations in word order reflecting syntactic differences between the two languages, and other equivalent variations in tone, formality, and complexity, that is, different yet comparable ways of saying the same thing (Table 1).

**Table 1.** Selected examples of acceptable differences.

Difference	Explanation
Original version: “such as drugs” Back translation: “like medicines”	The forward translation used the word “medicines” [medicinas] to capture the English term “drugs.” The term *medicina* can mean both “medicine” and “(legal/prescription) drug” in Spanish and is of equivalent reading level. (Conversely, the apparent cognate *droga* in Spanish—or “drug”—connotes an *illicit* drug, and thus would have been inappropriate to use in the Spanish translation, while the term fármacos has the intended connotation but is more complex than *medicinas*.)
Original version: “taking part” Back translation: “participation”	We deemed the Spanish forward translation use of “participation” [*participación*] to be acceptable because it is of similar reading level in Spanish, even though its cognate in English is not. *Participación* can equally be translated as “participation” and “taking part” in English.

Problematic discrepancies included variations between the original document and back translation that potentially altered the core or peripheral meaning of the content, including the omission or addition of key words or concepts and the use of terms that were not clear synonyms or were of nonequivalent registers (e.g. differences in tone, formality, or reading level).

The co-authors compared their coding to resolve classification discrepancies. They then focused on the problematic discrepancies, comparing each against the original English form, the forward translation (Step 1), and the back translation (Step 2) to determine where the issue arose. Errors related only to the back translation were disregarded for the remainder of the analysis since they did not reflect an actual issue with the Spanish translation.

*Step 4*—*Comparison of discrepancies related to the forward translation.* E.R. and K.M.B. reviewed each discrepancy that arose in the forward translation against the original English version to discuss ways in which the Spanish translation could be revised to convey the intended information more appropriately. They consulted with another member of the larger research team (native Chilean Spanish speaker) to discuss specific errors and get additional feedback on proposed revisions.

*Step 5*—*Revisions to the forward translations.* Based on the results of Step 4, E.R. revised each Spanish translation. E.R and K.M.B. then compared these translations to those produced by four professional translation firms, as described below.

### Description of translation evaluation process

*Step 6—Professional translations.* We hired four professional translation firms to produce independent English to Spanish translations of each of the three consent-related documents (generating a total of 12 documents, available upon request). We selected both general and research-specific firms to examine whether there were differences in translation quality. Two firms (A and D) specifically marketed themselves as specializing in medical and scientific translation. The other two firms (B and C) marketed themselves more broadly; however, Firm B included scientific translation within its scope and claimed to employ translators with a diverse range of research-area expertise. Firm C did not mention scientific translation as an emphasis or claim to have particular translator expertise.

*Step 7—Professional translations compared to research team’s translation.* We used the “compare” feature in Word to compare the 12 professional translations against the corresponding Spanish translation developed via Steps 1–5 by our research team. This resulted in 12 comparison documents, each with all changes marked to indicate differences between the professional translations and our own.

*Step 8*—*Comparison documents coded in NVivo 11.* The 12 comparison documents were uploaded to NVivo 11 for coding. One author (E.R.) evaluated each tracked change against the original English document and applied codes categorizing different types of errors using a standard rubric (Table 2).

**Table 2.** Primary qualitative codes.

Comparable reading level—Indicate cases in which a translation uses terms, phrases, or syntax of unequal reading level, regardless of directionality Broad understanding across dialects—Code cases in which a translation uses a term that is dialectic specific and unlikely to be widely understood by Spanish speakers of different Spanish dialects Equivalent voice—Code instances in which the translation changes the voice construction (from active to passive or passive to active), *only* in cases in which change in voice is *not* required to be syntactically correct Failure to specify—Translation lacks information critical to convey intended meaning Specify unnecessarily—Translation includes information that is not in the original translation *and* is unnecessary to convey intended meaning Mistranslation—The term/phrase in the translation does not have the same meaning as the term/phrase in English

*Step 9*—*Code review, resolution, and analysis.* K.M.B. independently reviewed each code report to ascertain agreement or disagreement with code application. K.M.B. and E.R. then met to discuss areas of disagreement. Because language is complex, contextually contingent, and subjective, there are often multiple more or less appropriate ways to communicate an idea. As such, we adopted a conservative approach to code errors, that is, both co-authors had to agree that a discrepancy was problematic (rather than simply a matter of preference) in order to code it as such. We then analyzed code reports to describe translation errors and to identify ways to improve our own materials.

*Step 10*—*Ease of readability measures.* We then reviewed each of the translations, our own and those from the professional firms (15 documents, in total) to assess reading ease and grade level using several variations of the Flesch Reading Ease score^28^ adapted for Spanish written materials, including the Fernández-Huerta index,^29^ which is one of the oldest and most commonly used readability formulas in Spanish.^30^

*Step 11*—*Development of final Spanish materials.* K.M.B. and E.R. compared variations between our own translation and those of the four professional firms (Steps 8 and 9) to further revise our materials.

## Results

We identified three basic types of translation errors: use of nonequivalent registers, errors of omission, and other mistranslations affecting meaning.

### Nonequivalent registers

The most common translation problem related to equivalency of register,^31^ specifically the appropriate use of language to capture the intended tone, formality, technicality, and comprehensibility of materials. Professional firms tended to replace less complex English terms with more advanced or technical terms in Spanish, despite the availability of comparable terms (Table 3). For example, one firm translated “heart disease” [*enfermedades del corazón*] as “cardiac diseases” [*enfermedades cardiacas*] (Firm A) and another as “coronary diseases” [*enfermedades coronarias*] (Firm C).

**Table 3.** Frequency of errors coded.

	Long form				Short form			
	Firm A	Firm B	Firm C	Firm D	Firm A	Firm B	Firm C	Firm D
Comparable reading level	34	56	27	77	15	19	9	29
Broad understanding across dialects	0	0	0	0	0	0	0	0
Failure to specify	0	1	6	5	0	0	3	0
Specify unnecessarily	2	7	13	2	0	0	0	0
Mistranslation	4	28	10	6	0	6	0	0
Parts of speech*	3	14	2	5	1	2	1	1
Total	43	106	60	95	16	27	13	30

* Verb tense, voice, and other syntactical errors.

As another example, there are various ways to translate “to go over” (in a statement about meeting with a health care provider to go over research results) into Spanish. Three translations (our own, Firm A, Firm B) used the verb “to review” [*revisar*], while two firms (C, D) used “to analyze” [*analizar*]. Both translations capture the semantic meaning of “to go over,” but the latter translation, which is a more complex term, may lose the pragmatic meaning. Table 4 provides additional examples of these kinds of non-equivalency issues.

**Table 4.** Examples of nonequivalent reading level.

English [with equivalent Spanish terms]	Spanish translation [with more technical/advanced terms]
This collection is called [*se llama*] the Duke Biobank	This collection is designated [*denomina*] the Duke Biobank (Firm A)
There will be a new consent form just for [*solo para*] these other studies	There will be a new consent form exclusively for [*exclusivamente para*] these other studies (Firms B/C)
We will ask you [*le pediremos/le preguntaremos*] for some basic information	We will solicit from you [*le solicitaremos*] some basic information(Firm D)
The risk of this happening is very small [*muy pequeño*]	The risk of this happening is very remote [*muy remoto*] (Firm B)
We will not give [*les daremos*] researchers your name.	We will not provide [*No proporcionaremos*] researchers your name. (Firm A)
… you may feel brief pain or have some bruising from the needle [*o la aguja puede dejarle un moretón*]	… it is possible that you may feel pain for a short time or that the needle can trigger/provoke a hematoma [*provoque un hematoma*](Firm D)
We encourage you [*invitamos*] to talk with your family and friends	We exhort/urge you [*Lo exhortamos*] to talk with your family and friends (Firm A)
Genes give the instructions for building the proteins that make our bodies work [*operar el cuerpo humano*]	Genes give the instructions for building the proteins that make the organism function [*el organismo funcione*] (Firm D)
No matter [*sin importar*] what you decide …	Independently of [*Independientemente de*] what you decide… (Firm D)
… some [*algo de/parte de*] of your blood	… a sample [*una muestra*] of your blood (Firm D)

The use of nonequivalent registers was not only limited to the use of more complex and scientific terms but also extended to sentence construction. Some professional firms had a tendency to combine and restructure content to produce longer, more complicated phrases and sentences. For example,

Original English: As the Biobank staff discusses this consent form with you, please ask him/her to explain any words or information that you do not clearly understand.

Professional Translation (Firm C): Given that the Biobank personnel will talk with you about this form, ask them to explain any term or information that you have not comprehended with clarity. [*Dado que el personal del Biobanco conversará con usted sobre este formulario de consentimiento, pídales que le expliquen cualquier término o información que no haya comprendido con claridad.*]

The use of more advanced technical terms, coupled with the use of longer, more complex syntactical constructions, contributed to variations in both short and long form readability (Table 5). Professional firms specializing in scientific and medical translation (Firms A and D) appeared to produce more readable materials than other firms.

**Table 5.** Readability.

Scale/index	Our translation	Firm A	Firm B	Firm C	Firm D
Short					
Fernández Huerta^29^	Average (63.1)	Average (62.5)	Somewhat difficult (58.4)	Somewhat difficult (57.3)	Average (61.3)
INFLESZ^46^	Average (58.4)	Average (57.7)	Somewhat difficult (53.1)	Somewhat difficult (52.5)	Average (56.2)
Legibilidad µ^47^	Difficult (48.1)	Difficult (48.3)	Difficult (45.5)	Difficult (45.1)	Difficult (46.2)
Grade level^48^	5.6	5.6	6.0	6.1	5.8
Long					
Fernández Huerta	Average (60.4)	Average (60.6)	Somewhat difficult (59.1)	Somewhat difficult (56)	Average (59.6)
INFLESZ	Average (55.9)	Average (55.6)	Somewhat difficult (54.1)	Somewhat difficult (51.4)	Average (54.6)
Legibilidad µ	Difficult (46.3)	Difficult (46.7)	Difficult (44.6)	Difficult (45)	Difficult (44.8)
Grade level	5.9	5.9	6.0	6.3	6.0
Quiz					
Fernández Huerta	Average (61.3)	Average (61.4)	Somewhat difficult (59.4)	Average (59.6)	Average (61.6)
INFLESZ	Average (56.4)	Average (56.3)	Average (54.5)	Average (54.7)	Average (55.8)
Legibilidad µ	Difficult (47.2)	Difficult (47.4)	Difficult (46.3)	Difficult (46.3)	Difficult (45.8)
Grade level	5.6	5.7	5.7	5.8	5.4

### Errors of omission

Professional firms commonly omitted words or phrases present in the original English forms. In many cases, omissions did not alter the pragmatic significance or core meaning of the material and thus are not described here, since the resulting translations were considered to be equally appropriate ways to convey the same material in Spanish.

However, a common omission that may lead to cultural incongruence was the common, though inconsistent, omission of the word “please” from translations. Less use of this deferential hedge could have a subtle effect on the overall tone of the consent materials (from more to less polite).

Other types of omissions may have the effect of reducing the clarity of study information. For example, one professional firm failed to re-identify the referent (genes) in a sentence, potentially diminishing participants’ understanding of the association between genes and responses to treatment:

Original English: Some of these studies may be about how genes affect health, or how genes affect response to treatment.

Professional Translation (Firm D): Some of these studies may be about how genes affect health, or the response to treatment. [*Algunos de estos estudios pueden ser sobre cómo los genes afectan la salud o la respuesta al tratamiento.*]

Some omissions decreased the amount of information participants would receive about certain aspects of the study; others actually altered the facts of the study (Table 6). In the most concerning case, one professional firm failed to specify that the Genetic Information Non-discrimination Act (GINA) makes it illegal for *health* insurance companies to discriminate against participants based on genetic information:

Original English: … that makes it illegal for health insurance companies, group health plans, and most employers to discriminate against you based on your genetic information.

Professional Translation (Firm C): … that makes it illegal for insurance companies, group health plans, and most employers to discriminate against you based on your genetic information. [*…. que prohíbe a las compañías de seguros, planes de salud grupales y a la mayoría de los empleadores discriminar a una persona por su información genética.*]

**Table 6.** Examples of errors of omissions.

Original English text [with omitted words/phrases]	Spanish translation [without omitted words/phrases]
We will replace this information with a code number [*un código numérico*]	We will replace this information with a code [*un código*](Firm C)
From this sample, the Biobank will be able to get things like plasma [*obtener cosas como plasma*], serum, blood cells, DNA, and RNA	From this sample, the Biobank will be able to get plasma, [*obtener el plasma*] serum, blood cells, DNA, and RNA(Firm D)
Some researchers will be from the United States, some may be from other countries around the world [*otros países del mundo*]	Some researchers will be from the United States, some may be from other countries [*de otros países*] (Firm D)
… that makes it illegal for health insurance companies, [*que compañías de seguros de salud*] group health plans, and most employers to discriminate against you based on your genetic information	… that makes it illegal for insurance companies, [*las compañías de seguros*] group health plans, and most employers to discriminate against you based on your genetic information (Firm C)
GINA also does not protect you against discrimination based on an already-diagnosed genetic condition or disease	It [*Tampoco lo*] also does not protect you against discrimination as a function of an illness or previously diagnosed genetic sickness (Firm B)
Researchers must study materials from many people over many years before they can know if the results have meaning. The results will not affect your care right now [*en este momento*]	Researchers must study materials from many people over many years before they can know if the results have meaning. The results will not affect your care [*no afectarán su atención médica*] (Firm C)

The omission of “health” from the translation results in an inaccurate description of GINA’s protections, which do not, for instance, protect against discrimination by life or disability insurance companies.

### Altered meaning

We identified a significant number of cases in which terms or concepts were imperfectly or imprecisely translated (Table 7). For example, translating the word “brief” in the English form to the word “mild” in the Spanish form or from the word “small” to “low.” Some of these mistranslations were more problematic than others. For instance, although the right to withdraw is fundamental to research ethics, one professional firm’s translation suggested that participants only have the right to stop temporarily (“interrupt participation”). As another example, some of our English materials included an introductory sentence, “You are being asked to …” However, two professional firms (Firm C and D) replaced “asked” with “invited.”“Ask” and “invite” represent different “speech acts” and thus “do” different things—entailing different role relations and obligations between the researcher and subject.^32^ The word “invite” is typically not used in consent forms because it introduces a sense of exclusiveness or allure to research participation.^33^

**Table 7.** Altered meaning.

Original English text [with same meaning]	Translation [with altered meaning]
You may feel brief pain [*un breve dolor*] or have some bruising	You may feel mild pain [*leve dolor*] or have bruises (Firm C)
We believe the chance these things will happen is very small [*muy pequeña*], but we cannot make guarantees	We consider the chance that this will happen to be very low [*muy baja*], but we can’t make guarantees (Firm D)
… you have the right to discontinue [*discontinuar*] participation at any time	… you have the right to interrupt [*interrumpir*] participation at any time (Firm A)
You are being asked [*está pidiendo*] to contribute a blood sample and health information to the Duke Biobank	We would like to invite you [*Quisiéramos invitarlo/Lo invitamos*] you to contribute a blood sample and health information to the Duke Biobank (Firm C/D)
Much of this research [*estas investigaciones*] is done using human tissue samples (such as blood) and health information	Much of this study [*esta investigación*] is done using human tissue samples (such as blood) and health information (Firm A)

## Discussion

As an increasing number of studies enroll non-English-speaking subjects, it is important to ensure the quality of translated materials for all participants. Issues have not only been reported in Spanish language translations^34^ but also in many diverse contexts^35^ and languages, including Navajo,^36^ Chinese,^37^ and Hindi.^26^

In this study, we sought to examine issues that can arise when consent materials are translated from English to Spanish. Drawing on expertise from five individuals associated with our research team, four of whom are native Spanish speakers of different dialects of Spanish, we crafted our own translations of consent materials using a rigorous, multi-step process. We then compared our translations to those produced by four professional translation firms to identify potential issues arising during the translation process.

We found three primary types of errors. In most cases, the errors were in the professional translations; however, we did, on occasion, identify errors within our own translations as well. Most commonly, professional firms replaced simpler terms in our original English documents with more technical and complex terms in their Spanish translations. This kind of error is particularly troubling when significant effort has been expended to simplify consent language and communicate more effectively. We also discovered errors of omission, in which the Spanish translations omitted substantive words or phrases from the original. Finally, we documented a range of mistranslations and imprecise translations that resulted in altered meaning.

Researchers should be aware of the varying range and degree of issues that inaccurate, imprecise, and culturally incongruent translations can introduce. Some of the translation-related errors we discovered, such as the omission of “health” from “health insurance” in describing specific legal protections provided by GINA, led to serious misstatements.

Other omissions, such as the routine deletion of “please” from consent materials, may have subtler, but nonetheless important effects on setting the overall tone of the study interaction. The impact and appropriateness of such omissions will vary by language and context. In some languages, for instance, the notion of “please” can be captured using other words or conveyed by other phrases, and thus, use of the term in consent materials may be redundant, awkward, or inappropriate; yet in other languages, including terms like “please” is necessary to convey politeness. Failing to set the proper tone can result in unintended consequences, such as subjects feeling disrespected or researchers being discredited.

Capturing the appropriate level of “deference and demeanor” in participant-facing materials requires a deep understanding of linguistic, contextual, and cultural norms and expectations.^38^ Thus, translators require not only “textbook” knowledge of both languages but also an appreciation of the social and cultural factors that affect how people interpret and understand meaning. To produce a quality translation, a translator must, at a minimum, have a well-developed understanding of the culturally meaningful variations in a language’s many constituent registers of linguistic usage.

Moreover, our findings highlight the importance of working with translators who have a basic understanding of research to accurately convey essential concepts, such as voluntariness and the right to withdraw. Although the professional firms specializing in medical and scientific translation (Firms A and D) produced more readable materials than the other firms (Table 5), specialization did not always result in fewer coding errors (Table 3). Based on these findings, we recommend that during the translation process researchers communicate with translators the meaning of key concepts to ensure they are precisely conveyed in translated materials.

Despite the complexities of translation and documented challenges in producing accurate materials, there are few if any regulatory standards to guide the translation process. Federal human subjects protections require only that information be presented to subjects in language understandable to them.^1,2^ A brief review of publicly available institutional review board (IRB) policies suggests wide variation in practice.^33,39–44^ Some IRBs, for example, require or recommend a back translation, while at least one actively discourages the step. Among those requiring back translation, some specify that the forward and back translations be conducted by two different individuals and/or that the person completing the back translation be blinded to the original English materials, while most IRBs have no such specifications.

Although IRBs requirements vary widely, most, at minimum, require that a translation be certified and that translators describe their qualifications. Yet it is unclear that either of these steps will guard against the types of errors we identified. With regard to certification, the American Translation Association describes a certified translation simply as one “accompanied by a signed statement attesting that the translation is accurate and complete to the best of the translator’s knowledge and ability … A translator does not need to be ‘certified’ in order to provide a ‘certified translation’.”^45^ With regard to qualifications, many IRBs consider native fluency to be sufficient proof of translator ability.^39–44^ Thus, we could have certified our own translation and satisfied many IRBs’ translation *and* translator requirements.

To the extent a research endeavor is committed to enrolling non-English speakers, commensurate resources are needed to produce high-quality translations. While our process was time intensive, it generated the most equivalent translation, in terms of tone, readability, and formality. For large research studies, this level of effort may be well worth it. In particular, when feasible, the following steps may help to improve the quality of translated materials:

- Seek to engage several individuals with native or advanced proficiency in both languages in developing and revising materials. Strive to identify translators who not only have intimate knowledge of both languages but who are also familiar with the goals for the ethical conduct of research, as well as principles and concepts of informed consent (e.g. the right to withdraw). Researchers who possess bilingual fluency may be best positioned to draft initial translations of the consent materials.
- Encourage the research team, regardless of linguistic ability, to take an active role in the translation process for their specific study. Researchers can, for example, communicate to the translator key aspects of the specific study, as well as important details about the translation (e.g. use neutral, simple language).
- Produce a back translation and carefully compare it to the original English materials. While many discrepancies identified in the back translation will not indicate errors in translation (i.e. are different yet equivalent ways of saying the same thing), a careful comparison can reveal subtle but problematic issues to be addressed. To take full advantage of the benefits of back translation, the person conducting it should neither have conducted the forward translation nor have seen the original English-language materials.
- Develop a systematic process to review the back translation and identify issues in the forward translation. We found the Word comparison feature to be a valuable tool to carefully and systematically examine differences between documents (e.g. original English and back translation documents).
- Conduct cognitive testing of the translated materials with native speakers to assess comprehensibility, clearness, and cultural appropriateness. (As part of our larger study on informed consent for biobanking, we conducted cognitive interviews using our translated materials; publication in process.)

Even when following all of these steps, there is no such thing as a perfect translation. In our own analysis, there were some instances in which the same English sentence was translated to Spanish using multiple equally appropriate, but different words or phrases. Indeed, there were instances in which we found a professional firm’s translation to be more accurate, more precise, or simply more readable than our own.

Given the subjective nature of language and the ability to communicate the same relative meaning in multiple ways, we used a conservative approach to identify issues with translations. Even so, the examples we highlight here illustrate *potential* issues; some Spanish speakers may disagree with our classification of particular translations as problematic. In addition, it is unclear from this evaluation the impact of translation errors. We cannot say, for example, the extent to which omitting “please” shifts the tone of the consent form in ways meaningful to all Spanish-speaking subjects or the use of more technical terms and complex phrases decreases subject comprehension; these are important areas for future research.

The subjectivity of any analysis of translated documents is perhaps less a limitation than an inevitability, given the subjectivity of language itself. It further illustrates the importance of adopting a thoughtful, rigorous approach to the development of translated materials—including, when possible, cognitive testing with native speakers. Careful translation and testing can help to ensure consent materials are comprehensible and convey the essential material prospective participants need to make informed decisions about research.
